# Dose Accuracy and Content Uniformity of Low-Dose Metoprolol Tablets: 3D Printing Compared with Tablet Splitting in Hospital Pharmacy Setting

**DOI:** 10.3390/pharmaceutics18050532

**Published:** 2026-04-27

**Authors:** Christine Larsen, Farnaz Shokraneh, Julius Lahtinen, Mahsa Bahman, Laura Mantila, Ludmila Hrižanovská, Niklas Sandler

**Affiliations:** 1 Mayo Clinic, Pharmacy Services, 200 First Street SW, Rochester, MN 55905, USA; larsen.christine@mayo.edu; 2 CurifyLabs Oy, Salmisaarenaukio 1, 00180 Helsinki, Finland; farnaz.shokraneh@curifylabs.com (F.S.); julius.lahtinen@curifylabs.com (J.L.); mahsa.bahman@curifylabs.com (M.B.); laura.mantila@curifylabs.com (L.M.); ludmila.hrizanovska@curifylabs.com (L.H.); 3 Pharmaceutical Sciences Laboratory, Åbo Akademi University, 20520 Turku, Finland

**Keywords:** metoprolol tartrate, tablet splitting, 3D printing, hospital pharmacy, dose accuracy, content uniformity, compounding

## Abstract

**Background**: Tablet splitting is widely used to individualize dosing of metoprolol tartrate when low-dose formulations are unavailable. However, splitting may lead to variability in dose accuracy, which can be clinically relevant at low dose strengths. This study compared the accuracy and precision of manual tablet splitting versus automated 3D printing for producing low-dose metoprolol tartrate tablets in a hospital setting. **Methods**: Commercial 25 mg metoprolol tartrate tablets from two manufacturers were manually split at Mayo Clinic into 12.5 mg halves and 6.25 mg quarters following established procedures. CurifyLabs’ Compounding System, including the Pharma Printer and CuraBlend^®^-based formulations, was used at two hospital pharmacy sites and a compounding pharmacy to produce 6.25 mg and 12.5 mg tablets via 3D printing. Mass variation and content uniformity were evaluated using USP <905> criteria and validated HPLC methods. **Results**: Manually split tablets showed high variability, with 6.25 mg quarters ranging from 24.9% to 142.8% API content and 12.5 mg halves from one manufacturer averaging only 66.6%, indicating frequent underdosing. In contrast, 3D-printed tablets achieved mean API contents within 90–103%, standard deviations below 5%, and acceptance values under 15 across all sites and formulations. **Conclusion**: Manual tablet splitting may not reliably meet pharmacopeial expectations for dose accuracy and content uniformity at lower dose strengths. Automated 3D printing produced consistent low-dose tablets and may offer a validated alternative where available.

## 1. Background

Tablet splitting is a widespread practice used to adjust doses, reduce medication costs, and facilitate swallowing, especially in pediatric, geriatric, and polymedicated populations [1,2]. This approach is particularly common for drugs that lack commercially available low-dose formulations or for patients who require individualized dosing regimens. Despite its ubiquity, tablet splitting is fraught with challenges that undermine dose precision and therapeutic efficacy. Numerous studies have documented substantial variation in both the weight and drug content of split tablets, often exceeding pharmacopeial thresholds and regulatory guidelines [3,4].

A core concern in tablet splitting lies in its inability to ensure dose uniformity, particularly for drugs with narrow therapeutic indices (NTIs) such as metoprolol tartrate. Metoprolol, a selective β1-adrenergic blocker, is widely used in the management of hypertension, angina pectoris, arrhythmias, and heart failure, where precise dose titration is crucial for efficacy and safety [5]. The clinical consequences of inaccurate dosing are significant: subtherapeutic doses may result in inadequate blood pressure or heart rate control, while supratherapeutic doses can lead to adverse effects such as bradycardia, hypotension, or even heart block [6]. In a landmark study, Polli et al. [7] reported that a significant proportion of manually halved tablets failed to meet the FDA’s uniformity criteria, with some half-tablets containing less than 70% or more than 130% of the labeled dose. Similarly, Helmy [4] assessed 16 commonly used medications and found that over half of the split units failed to comply with acceptable weight variation limits. Ciavarella et al. (2016), applying FDA scoring guidelines, showed that scored tablets did not consistently meet content uniformity requirements post-splitting, raising serious concerns regarding their use in dose adjustment [8].

The problem is exacerbated when very low doses are required, such as the 6.25 mg dose of metoprolol tartrate often needed for titration in heart failure or pediatric patients. Deviation from the target dose in these cases can result in subtherapeutic effects or adverse reactions [9]. This issue is further intensified when tablets are quartered, as shown in studies by Van Santen et al. (2002) and Madathilethu et al. (2018), where more than 30% of quartered fragments were outside acceptable mass ranges [10,11]. Notably, the FDA has recognized these risks and issued guidance emphasizing that only tablets specifically designed and tested for splitting should be considered for this practice [12].

Operational inefficiency is another significant drawback of manual tablet splitting. The process is time-intensive, operator-dependent, and exposes patients to significant inter-dose variation [2,8,10,13]. In hospital and pharmacy settings, manual splitting not only increases the risk of dosing errors but also adds to the workload of healthcare professionals [14]. Furthermore, the lack of standardization in splitting techniques, variability in tablet hardness, and the presence or absence of score lines all contribute to inconsistent outcomes [10].

To overcome these limitations, there is a growing interest in automated and digital compounding technologies. 3D printing has emerged as a transformative technology in pharmaceutical compounding, offering a pathway to individualized therapy with milligram-level accuracy [15,16,17,18]. 3D printing enables the fabrication of unit-dosed oral solid formulations that precisely match patient-specific dosing needs, thereby bypassing the need for tablet splitting altogether [19,20,21]. Recent studies have demonstrated the superiority of 3D-printed tablets over traditionally split tablets in terms of content uniformity and dose accuracy [16,17,18,22]. The reproducibility across decentralized sites further confirms that automated compounding systems can maintain quality standards independent of production location [16,23,24].

**Figure 1:**
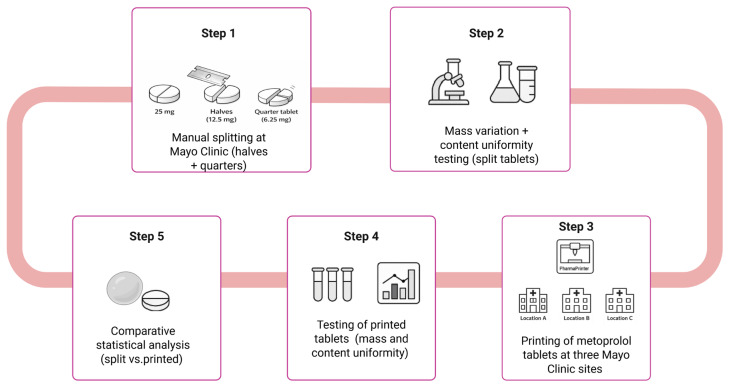
Study workflow for comparative evaluation of split versus 3D-printed metoprolol tartrate tablets. The workflow illustrates the two production pathways (manual splitting at Mayo Clinic and 3D printing via the CurifyLabs Compounding System Solution) and the analytical steps applied to each.

The present study demonstrates the use of an automated semi-solid extrusion-based 3D printing platform for the individualized compounding of metoprolol tartrate tablets in clinically relevant low doses (6.25 mg and 12.5 mg). Using a validated gel-based excipient matrix (CuraBlend^®^ gel tablet) and an anhydrous troche base evaluated for content uniformity, we compare the performance of printed versus split tablets in terms of mass variation and content uniformity across decentralized hospital sites (Figure 1).

## 2. Materials and Methods

### 2.1. Materials

For the automated dosing experiments, excipient bases formulated with metoprolol tartrate (sourced from Caesar & Loretz GmbH, Hilden, Germany) and Polysorbate 80 (Caesar & Loretz GmbH, Hilden, Germany) were employed. Two different pharmaceutical excipient bases under the CuraBlend^®^ brand, supplied by CurifyLabs (Helsinki, Finland), were used in the formulation development. The CuraBlend^®^ gel tablet base comprised predominantly purified water, gelatin, and cocoa butter, resulting in a soft, chewable matrix suitable for immediate-release applications and pediatric dosage forms. The CuraBlend^®^ troche excipient base is a polyethylene glycol-based mixture providing a water-free matrix.

For analytical procedures, HPLC-grade Acetonitrile was sourced from Fisher Scientific (Loughborough, UK). All other chemicals and reagents were of analytical grade. In the blister packaging process, 3/16″ Mini Medi-Cup^®^ Plus™ Blisters (MD425, MediDose Group, Ivyland, PA, USA) were used for packaging both gel tablets and anhydrous troches. Additionally, sterilized single-use PVC syringes (100 mL) with Luer-lock fittings (CurifyLabs, Helsinki, Finland) were utilized during the printing process.

Commercial metoprolol tartrate 25 mg tablets were obtained and pre-split by the clinical team at Mayo Clinic (Rochester, MN, USA) using standard hospital protocols. Two strength variants were prepared: 12.5 mg (halved from 25 mg units) and 6.25 mg (quartered). Tablets from two manufacturers were included: Tablets A (MYLAN/Viatris, Canonsburg, PA, USA) and Tablets B (Rising Pharma, East Brunswick, NJ, USA). Both commercial products were supplied in the manufacturers’ original packaging (HDPE bottles with induction-sealed closures) and stored per the package inserts at controlled room temperature (20–25 °C, with excursions permitted to 15–30 °C), protected from light and moisture, until the splitting event. After splitting, halves and quarters were transferred to sealed, labelled containers for shipment. Split tablets were shipped to CurifyLabs’ quality control laboratory in Helsinki, Finland, for analysis under controlled ambient conditions during transit.

### 2.2. Methods

#### 2.2.1. Development of Formulation and Process Validation

CurifyLabs developed and validated personalized metoprolol tartrate gel tablet formulations using its proprietary CuraBlend^®^ base and compounding technologies. Three concentrations (0.5%, 2%, and 3% *w*/*w*) were prepared to support flexible dosing needs. Each formulation underwent internal process validation to ensure suitability for semi-solid extrusion and 3D printing.

CurifyLabs’ Compounding System Solution (CSS) is an advanced, modular platform designed to support non-sterile pharmaceutical compounding in a decentralized yet standardized manner. It integrates pharmaceutical-grade excipient bases, proprietary compounding software with an embedded formulation library and order management, and automated hardware including a 3D printer with semi-solid extrusion capability and a planetary mixing unit, along with primary packaging systems and integrated quality control. The CSS enables on-demand preparation of personalized medicines with digital traceability, batch documentation, and real-time monitoring. Each unit dose is subject to 100% weight control using a precision scale.

Metoprolol tartrate formulations were prepared using the CuraBlend^®^ excipient base with the following compositions: 0.5% formulation (0.5% *w*/*w* API, 1.0% *w*/*w* Polysorbate 80, 98.5% *w*/*w* excipient base); 2% formulation (2.0% *w*/*w* API, 2.0% *w*/*w* Polysorbate 80, 96.0% *w*/*w* excipient base); and 3% formulation (3.0% *w*/*w* API, 3.0% *w*/*w* Polysorbate 80, 94.0% *w*/*w* excipient base). Each formulation was homogenized using a planetary mixer (PM 140, Gako Deutschland GmbH, Scheblitz, Germany) at 2800 rpm for 10 min, then transferred into sterilized, single-use 100 mL PVC syringes with Luer-lock connectors.

Automated compounding was carried out using the CurifyLabs Pharma Printer, an extrusion-based 3D printer optimized for semi-solid formulations. Formulation-specific parameters were configured via the system’s software (version 3.0.63) and stored in the formulation library, enabling precise and flexible production with digitally logged batch records [15].

The printing process is classified as semi-solid extrusion (SSE)-based additive manufacturing. During production, the CuraBlend^®^ formulation is extruded as a controlled mass directly into individual Medi-Cup^®^ blister cavities from a Luer-locked syringe under precise positional and gravimetric control. Each unit dose is individually dispensed under digital batch control with 100% in-line weight verification; there is no bulk pour, no shared mold cavity, and no post-formation trimming. This distinguishes the process from conventional mold-casting, in which a bulk preparation is poured into a multi-cavity mold and allowed to set before demolding. The layer-by-layer deposition, individual weight feedback, and single-cavity filling are characteristic of additive manufacturing and are essential for the dose accuracy reported in this study.

Two categories of printed formulations are reported in this study. Development formulations (0.5%, 2%, and 3% *w*/*w* metoprolol tartrate in CuraBlend^®^ gel tablet base, 400 mg unit weight) were used for initial process validation, dissolution characterization, and stability testing (Section 3.1 and Section 3.2). A separate set of comparator formulations (4.0% *w*/*w* metoprolol tartrate in both CuraBlend^®^ gel tablet and CuraBlend^®^ troche bases) was produced specifically for the head-to-head comparison with split commercial tablets. Target unit weights for the comparator batches were 156 mg (6.25 mg dose) and 313 mg (12.5 mg dose). Table 1 provides a complete formulation and batch summary for all comparator conditions.

#### 2.2.2. In Vitro Dissolution Testing

Dissolution testing was conducted using a USP Apparatus 1 (basket) apparatus (DT 128 light; ERWEKA, Heidenstam, Germany), operated at 100 rpm and maintained at 37 ± 0.5 °C. Six individual tablets were each placed into separate vessels containing 500 mL of deionized water; sink conditions were confirmed based on the solubility of metoprolol tartrate in water at 37 °C, which greatly exceeds the tested concentrations. At predetermined time intervals—5, 10, 15, 20, 30, 45, and 60 min—1 mL aliquots were withdrawn and an equal volume of fresh deionized water replenished to maintain sink conditions. Samples were filtered through 0.2 μm regenerated cellulose syringe filters prior to HPLC analysis.

#### 2.2.3. Stability Study

Stability testing was conducted on 3D-printed metoprolol tartrate tablets at 0.5%, 2%, and 3% concentrations in CuraBlend^®^ excipient bases. Stability assessments included assay (n = 6), pH measurement, and physical appearance. Samples were stored at 25 ± 2 °C/60 ± 5% RH in sealed Medi-Cup^®^ blister packaging (primary container). Prespecified acceptance criteria were as follows: assay 90–110% of label claim, pH 4.0–6.0, and no visible change in color or consistency. Sampling time points were t = 0, 1, 3, 6, and 9 months for the 3% formulation, t = 0, 1, 3, and 6 months for the 2% formulation, and t = 0, 1, and 3 months for the 0.5% formulation. Full individual time-point data are provided in Supplementary Table S2.

#### 2.2.4. Splitting of Tablets

Tablet splitting was performed to reflect routine hospital pharmacy practice. All tablet subdivision was carried out by a single pharmacy technician with standard hospital pharmacy training and experience, including routine tablet splitting as part of medication preparation and dispensing workflows. The technician’s work was checked and validated by a single pharmacist with standard hospital pharmacy training and experience. All halves and quarters were produced in one continuous preparation event; in total, 95 halves and 95 quarters were created for each of Products A and B (4 × 95 = 380 samples total).

Prior to study initiation, both operators followed a standardized tablet-splitting approach to promote consistency across preparations. Standardization focused on tablet positioning, alignment with the manufacturer’s score line, and consistent handling of split portions. No study-specific optimization or precision-enhancing techniques beyond routine practice were employed.

Tablet splitting was performed manually using a single-edge razor blade aligned with the tablet score line. Intact tablets were placed on a clean, flat surface, and downward pressure was applied to divide the tablet along the score. Tablet halves were generated first. Tablet quarters were produced by halving previously generated halves, consistent with common hospital pharmacy practice when quarter doses are required. Commercial tablet-splitting devices were not employed, as such devices do not reliably allow for precise alignment of the splitting mechanism along manufacturer-scored lines, particularly when generating tablet quarters. Manual alignment using a razor blade was therefore selected to better reflect routine institutional practice and to allow consistent score-line positioning.

Following splitting, both the pharmacy technician and pharmacist visually inspected all split portions to confirm that the final products were approximately one-half or one-quarter of the intact tablet, as appropriate. This visual inspection reflected standard practice and did not include weighing or mass-based adjustment of split units. Fragments or powder generated during splitting were not collected, recombined, or otherwise corrected for, as this would not reflect routine handling in clinical practice. Visually intact halves and quarters were retained for further testing. Split portions that exhibited gross mechanical failure (e.g., fragmentation into multiple unusable pieces) were excluded from analysis; no exclusions were based on mass, symmetry, or appearance beyond practical usability.

This tablet-splitting methodology was selected to represent common hospital pharmacy practice, where subdivision of commercial tablets is frequently performed manually by trained personnel under time and workflow constraints. The intent of this comparator arm was to reflect real-world institutional practice, including inherent sources of variability, rather than to demonstrate best-case tablet-splitting performance.

#### 2.2.5. Evaluation of Split Tablets

Each split tablet batch was subjected to mass variation testing according to USP <905> and Ph. Eur. 2.9.5, and content uniformity testing via validated HPLC assay according to Ph. Eur. 2.9.40 and USP <905>.

For mass variation, 95 tablets from each dose and manufacturer were individually weighed using a calibrated analytical balance. The percentage deviation of each split tablet unit was calculated both relative to the group mean and relative to the theoretical half- or quarter-tablet target mass derived from the mean intact tablet weight and compared with the ±10% pharmacopeial threshold. For content uniformity, 30 units from each group were randomly selected from the pool of halves or quarters produced during the preparation event and assessed for API uniformity using a validated HPLC method; HPLC analysts were blinded to dose strength and manufacturer, with samples identified only by an anonymized batch code. Each split unit was fully dissolved prior to HPLC analysis, with complete dissolution verified visually before filtration.

Metoprolol tartrate assay and content uniformity analyses were performed using an HPLC system (Thermo Scientific™ Vanquish, Germering, Germany) with the Chromeleon™ (version 7.3.2) Chromatography Data System software. The system was equipped with a C18 column (4.6 × 100 mm i.d., 2.5 μm particle size, VanGuard FIT, Wilmslow, UK) and Diode Array Detectors. The mobile phase consisted of a gradient of 20 mM potassium phosphate dibasic buffer and acetonitrile at a flow rate of 0.7 mL/min, injection volume 5 μL, detection at 225 nm, column temperature 25 °C, and total run time 9 min.

For standard preparation, 25 mg of metoprolol tartrate was dissolved in 50 mL of deionized water (500-ppm stock solution). Working standards within the validated linearity range of 70–130 ppm were prepared by dilution. For sample preparation, each tablet was dissolved in 50–100 mL deionized water (volume based on strength), heated at 50 °C until fully dissolved, cooled, vortexed, and filtered through 0.2 μm syringe filters (MontaMil^®^, Frisenette ApS, Knebel, Denmark) before HPLC analysis.

The HPLC assay method was validated in accordance with ICH Q2(R2) and USP <1225> guidelines. Specificity was confirmed by placebo interference testing, with no peak observed at the analyte retention time (5.05 min); resolution 5.81, tailing factor 1.5, theoretical plates > 111,000. Linearity was established over 70–130 ppm (r^2^ = 0.9984; y-intercept ≤ 5% of target area). Accuracy (recovery) was 100.1% at 70% level (RSD 0.7%), 99.8% at 100% (RSD 0.5%), and 97.9% at 130% (RSD 0.1%). Repeatability (intra-day) at 100 ppm gave RSD ≤ 0.64% across three analysts; intermediate precision (inter-day, two days) yielded RSD ≤ 2.0% with mean concentration difference between days ≤ 0.6%. Solution stability showed ≤ 1.4% change over 48 h. Robustness was demonstrated across ±10% flow-rate variation, column temperature (40 °C), alternative column (VanGuard FIT), and pH range 8.9–9.3. LOD was 0.05 ppm (S/N ≥ 761) and LOQ 0.5 ppm (S/N ≥ 6136, RSD 1.08%). The full validation report is provided as Supplementary Material Table S4.

Acceptance Value (AV) was calculated in accordance with Ph. Eur. 2.9.40 and USP <905>: AV = |M − $\bar{X}$| + ks, where M equals $\bar{X}$ if $\bar{X}$ is between 98.5 and 101.5 (otherwise 98.5 or 101.5), k = 2.4, and s = standard deviation. The AV must not exceed 15 to meet acceptance criteria.

For split tablets, n = 30 units per group were assayed to support the Ph. Eur. 2.9.40/USP <905> content-uniformity Level 1 + Level 2 decision framework, given the high expected variability of manual subdivision. For 3D-printed tablets, n = 10 per batch corresponds to the Level 1 sample of the same pharmacopeial framework and was pre-specified based on the 100% in-process weight control of every printed unit. The identical acceptance-value calculation (AV ≤ 15, k = 2.4) was applied to both arms.

#### 2.2.6. Transfer of the Automated Compounding System Solution Technology

CurifyLabs CSS technology was installed at two Mayo Clinic hospital pharmacy sites and a compounding pharmacy (Location A, Location B, and Location C), including a planetary mixer, Pharma Printer, and software with the formulation library. Pre-validated CuraBlend^®^ gel tablet formulations of metoprolol tartrate were produced at each site, with a minimum of 25 tablets per dose strength. CuraBlend^®^ troche formulations (4.0% *w*/*w* metoprolol tartrate) were also produced at all three sites for content uniformity testing to demonstrate platform versatility with a second excipient base.

#### 2.2.7. Testing of 3D-Printed Tablets

Printed tablets (both gel and troche forms) were collected from each site for comparative analyses and stored under controlled conditions during transit. For mass variation, 20 printed tablets per batch were weighed against the ±10% acceptance criteria. For content uniformity, n = 10 tablets per dose strength per site were dissolved and analyzed by HPLC following the same procedures as for split tablets.

The experimental unit for 3D-printed tablets was the individual batch. One independent batch per site per dose strength per excipient base was produced (3 sites × 2 dose strengths × 2 bases = 12 batches total). Mass variation (n = 20 tablets) and content uniformity (n = 10 tablets) were assessed within each batch. Because only a single batch per condition was produced, individual tablets within a batch cannot be treated as independent replicates for between-batch inference, and the analyses should therefore be interpreted as descriptive at the batch level with supportive inferential comparisons at the tablet level (see Section 2.2.8).

#### 2.2.8. Comparative Evaluation Strategy

The comparative analysis focused on dosing accuracy (measured API content vs. theoretical target), precision and content uniformity (SD and AV), and manufacturing reproducibility across decentralized sites. Data were stratified by production site, dose strength, formulation base, and source (split vs. 3D-printed). The primary inferential comparison (Section 3.5.1 and Section 3.5.2) used the CuraBlend^®^ gel tablet as the pre-specified printed formulation matched to the split-tablet comparator. Troche data are presented descriptively in Section 3.4 and Table 2 to demonstrate that the platform also meets pharmacopeial criteria with a second excipient base; troche results are not included in the split-versus-print inferential contrast.

Statistical analyses were performed using IBM SPSS Statistics (version 30.0.0.0). Because only one batch (or one splitting session) was produced per condition, the primary analysis is descriptive: mean, SD, minimum, maximum, and acceptance value (AV) were calculated per subgroup, and pharmacopeial pass/fail criteria (AV < 15) were applied at the individual-batch level. As a supportive analysis at the tablet level, Levene’s test assessed homogeneity of variances, independent-samples t-tests (or Welch’s t-test where variances were unequal) compared pairs of groups, and one-way ANOVA with Games–Howell post hoc testing was used for comparisons involving more than two groups (α = 0.05). These inferential results should be interpreted with the caveat that tablets within a single batch are not fully independent observations, and the *p*-values therefore relate to within-batch tablet-level variation rather than to batch-to-batch reproducibility.

## 3. Results

### 3.1. Development of Formulation and Process Validation

Three development formulations using CuraBlend^®^ gel tablet base were developed, incorporating metoprolol tartrate at concentrations of 0.5%, 2%, and 3% *w*/*w*, each with polysorbate (1–3%) to enhance uniform dispersion and extrusion performance. Tablets were manufactured in 400 mg unit weights, designed to deliver 2 mg (0.5%), 8 mg (2%), or 12 mg (3%) of metoprolol tartrate per tablet, suitable for personalized pediatric and geriatric dosing. Internal process validation confirmed printability, mass uniformity, and content uniformity within USP limits. All dosage forms were packed in Medi-Cup^®^ blisters under controlled ambient conditions with digitally logged batch-specific data. These development formulations were used for dissolution characterization and stability testing (Section 3.2) and are distinct from the 4.0% *w*/*w* comparator formulations used in the head-to-head comparison with split tablets (see Table 1 and Section 3.4 and Section 3.5).

### 3.2. Drug Release and Stability Studies

In vitro dissolution testing confirmed immediate-release behavior across all three gel tablet formulations, with over 50% drug dissolved within 5 min for the 3% (51.5%) and 0.5% (46.4%) formulations, while the 2% formulation showed slightly slower onset (36.8%). Complete or near-complete release (>100%) was observed across all formulations by 30 min. Full dissolution profile data for each formulation are provided in Supplementary Table S5.

Stability testing confirmed that the 2% and 3% formulations maintain acceptable assay values (within 99–104%) and stable pH (4.6–5.1) under ambient conditions for at least 3 months (2% formulation) and 9 months (3% formulation), respectively (full time-point data in Supplementary Table S2). The 0.5% formulation showed a decline in assay exceeding 5% loss by the third month, falling outside ICH Q1A(R2) acceptability limits. These results confirm that the 2% and 3% formulations are chemically stable and suitable for clinical use over a longer period, while the 0.5% formulation demonstrates a shorter shelf-life.

### 3.3. Mass Variation and Content Uniformity of Commercially Split Tablets

**Figure 2:**
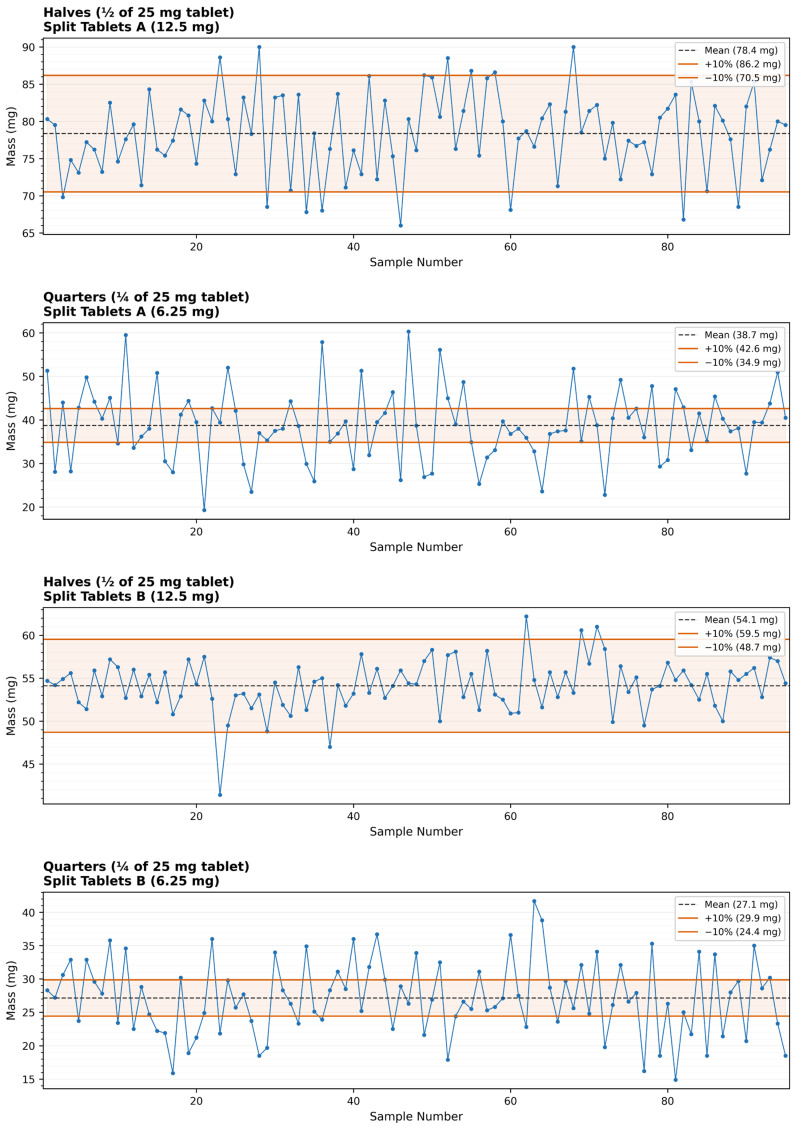
Mass variation of split commercial metoprolol tartrate tablets prepared as halves (12.5 mg) and quarters (6.25 mg) from Manufacturers A and B. Box plots show the distribution of tablet weights; horizontal lines indicate the median, boxes indicate interquartile range, and whiskers represent the full data range. Solid lines indicate the ±10% pharmacopeial acceptance limits. Raw individual-tablet mass data are provided in Supplementary Table S1.

The mass variation analysis revealed significant inconsistencies in tablet weights across both manufacturers and dose strengths. Tablets A 6.25 mg showed the greatest deviation, with a mean weight of 38.7 mg (SD 8.5 mg; range 19.3–60.3 mg). Tablets B 6.25 mg demonstrated a lower mean of 27.2 mg (SD 5.5 mg; range 14.9–41.7 mg). For the 12.5 mg split tablets, Tablets A showed a mean weight of 78.3 mg (SD 5.6 mg; range 66.0–90.0 mg), while Tablets B averaged 54.1 mg (SD 3.0 mg; range 41.4–62.2 mg). As per USP guidelines, tablets with a target weight below 135 mg allow a maximum deviation of ±10% from the mean (Figure 2).

To complement the group-mean analysis, mass deviation was also evaluated against the theoretical half- or quarter-tablet target mass, estimated as one-half or one-quarter of the mean intact tablet weight (Tablets A: 156.8 mg; Tablets B: 108.2 mg; intact weights estimated from mean half-tablet weight × 2, as intact tablets were not individually weighed). When assessed against these theoretical targets, 59.4% (60/101) of Tablets A quarters and 61.5% (64/104) of Tablets B quarters exceeded the ±10% pharmacopeial threshold (SD of deviations: 22.4% and 21.4%, respectively). In contrast, only 16.5% (17/103) of Tablets A halves and 7.1% (7/98) of Tablets B halves fell outside the ±10% limit (SD: 7.4% and 6.2%, respectively). A modest systematic mass loss of −1.3% was observed for Tablets A quarters relative to the theoretical quarter-target, consistent with material loss during the additional splitting step required for quartering.

**Figure 3:**
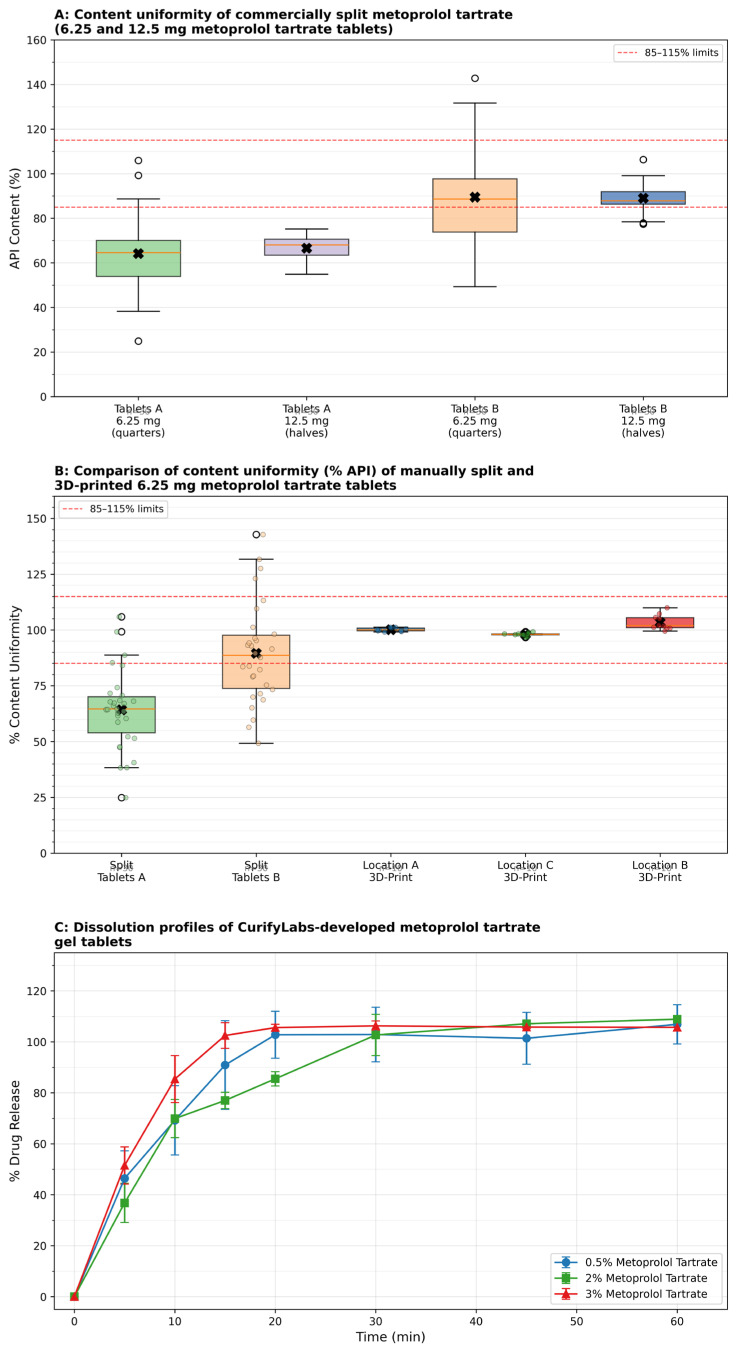
Quality evaluation of metoprolol tartrate tablets prepared by manual splitting and 3D printing: content uniformity and dissolution performance. (**A**) Content uniformity of split commercial tablets (Manufacturers A and B) at 6.25 mg and 12.5 mg. (**B**) Comparison of 6.25 mg content uniformity across manual splitting and 3D printing at Locations A–C. (**C**) Dissolution profiles of CurifyLabs-developed metoprolol tartrate gel tablet formulations (0.5%, 2%, and 3%) over 60 min. Raw individual API-content and mass data for all conditions shown in panels (**A**,**B**) are provided in Supplementary Table S1.

Content uniformity results further demonstrated the limitations of tablet splitting (Figure 3, Panel A). Tablets A 6.25 mg (quartered) showed poor content uniformity with a mean API content of only 64.2% (range 24.9–105.9%; SD 17.6%), indicating that many doses fell significantly below the acceptable therapeutic range. Tablets A 12.5 mg (halved) had a mean of 66.6% (range 54.9–75.2%), suggesting systemic underdosing. Tablets B results, although closer to the intended dosage on average, still exhibited significant variability: the 6.25 mg quarters had a mean of 89.5% but with extreme variability (SD 22.5%, range 49.3–142.8%), while the 12.5 mg halves showed a mean of 89.0% (SD 6.2%, range 77.4–106.3%). These findings reflect how splitting—especially quartering—amplifies variability and compromises dose accuracy.

### 3.4. Technology Transfer and On-Site Production at Mayo Clinic Locations

The technology transfer of CurifyLabs’ CSS platform to three Mayo Clinic locations enabled local on-demand printing of 6.25 mg and 12.5 mg metoprolol tartrate tablets using the CuraBlend^®^ gel tablet base. At Location A, the 6.25 mg tablets showed mean API content of 100.2% (SD 0.77%, AV 2) and 12.5 mg tablets a mean of 100.0% (SD 2.76%, AV 7). At Location B, the 6.25 mg tablets showed mean 103.4% (SD 3.26%, AV 10) and 12.5 mg tablets mean 101.3% (SD 0.55%, AV 1). Location C produced 6.25 mg tablets with mean 98.0% (SD 0.79%, AV 2) and 12.5 mg tablets with mean 98.9% (SD 2.23%, AV 5). All batches met pharmacopeial specifications (AV < 15), as summarized in Table 2.

Troche-base formulations of the same dose strengths were also produced at all three sites using the Pharma Printer platform. At Location A, the 6.25 mg troche tablets achieved mean 96.9% (SD 5.43%, AV 14) and 12.5 mg mean 98.2% (SD 3.10%, AV 8). Location B reported 6.25 mg mean 96.6% (SD 4.24%, AV 12) and 12.5 mg mean 93.7% (SD 1.44%, AV 8). Location C produced 6.25 mg troche tablets with mean 90.3% (SD 2.43%, AV 13) and 12.5 mg with mean 95.1% (SD 2.54%, AV 9). All printed tablets met pharmacopeial specification, verified by 100% weight control on each individual unit.

Using the CuraBlend^®^ gel tablet base, the 6.25 mg and 12.5 mg doses had target weights of 156 mg and 313 mg, with average measured weights of 157.0 ± 3.3 mg and 314.0 ± 5.8 mg, respectively. For the anhydrous troche base, the 6.25 mg dose averaged 159.3 ± 2.5 mg and 153.3 ± 6.4 mg, and the 12.5 mg dose averaged 314.7 ± 7.3 mg and 318.0 ± 6.9 mg across sites, all within specified limits.

### 3.5. Comparative Evaluation: Manual Splitting vs. Automated Compounding

#### 3.5.1. Content Uniformity of 6.25 mg Metoprolol Tartrate Tablets

A one-way ANOVA across five groups (Tablets A split, Tablets B split, and 3D-printed from Locations A, B, and C) revealed a statistically significant difference (F(4, 85) = 18.73, *p* < 0.001; ω^2^ = 0.441). Levene’s test was significant (*p* < 0.001), so Games–Howell post hoc testing was applied. Tablets A showed the lowest and most variable content uniformity (mean 64.2%, SD 17.6%), significantly lower than all three 3D-printed groups (*p* < 0.001 for each). Tablets B (mean 89.5%, SD 22.5%) still differed significantly from the Location B printed group (*p* = 0.019). All three 3D-printed groups—Location A (100.2%, SD 0.77%), Location B (103.4%, SD 3.37%), and Location C (98.0%, SD 0.79%)—were within pharmacopeial content uniformity limits (85–115%) with minimal variability (Figure 3, Panel B).

#### 3.5.2. Content Uniformity of 12.5 mg Metoprolol Tartrate Tablets

A similar pattern was observed for 12.5 mg tablets: ANOVA revealed highly significant differences (F(4, 85) = 167.41, *p* < 0.001; ω^2^ = 0.881). Games–Howell post hoc testing confirmed that Tablets A had significantly lower mean content uniformity (66.6%, SD 5.9%) than all 3D-printed groups (*p* < 0.001). Tablets B (89.1%, SD 6.2%) also differed significantly from each printed group. The three 3D-printed batches—Location A (100.0%, SD 2.8%), Location B (101.3%, SD 0.5%), and Location C (98.9%, SD 2.2%)—showed tightly clustered means well within the pharmacopeial range. Together, these results demonstrate that, under the conditions tested, the 3D-printed tablets achieved higher dose precision and consistency than the manually split tablets. The dissolution profiles of the three development formulations (0.5%, 2%, and 3% *w*/*w*) are presented in Figure 3, Panel C.

## 4. Discussion

This study highlights the critical limitations of manual tablet splitting for dose individualization and the significant advantages offered by automated 3D printing for compounding precise low-dose metoprolol tartrate formulations.

The results clearly demonstrated that manually split tablets failed to achieve acceptable content uniformity, especially for quartered 6.25 mg doses. Tablets A quarters showed a mean API content of 64.2% with an extreme range from 24.9% to 105.9%, while Tablets B quarters, despite a higher mean of 89.0%, also exhibited unacceptable variability ranging up to 142.8%. This is consistent with earlier findings where splitting into quarters resulted in dose deviations far exceeding pharmacopeial specifications [11]. Even halved tablets (12.5 mg) were suboptimal, with Tablets A halves averaging 66.6% API content, indicating systemic underdosing rather than random error. Such variability is clinically significant for metoprolol tartrate, a drug with a narrow therapeutic index [9,25].

The variability observed in split tablet halves and quarters can be attributed to several well-described factors inherent to manual tablet subdivision. Mechanical fracture along scored lines is influenced by tablet hardness, shape, and score depth, which can result in uneven mass distribution even when tablets are nominally scored. Manual splitting also introduces unavoidable powder loss and fragmentation, which are not readily recoverable in routine practice and may further contribute to dosing variability. In addition, variability may arise from operator-dependent factors such as visual alignment of the score line and handling of split portions, despite standardized technique and pharmacist verification. These sources of variability represent intrinsic limitations of tablet splitting in clinical practice and are consistent with prior reports describing poor dose uniformity following subdivision of commercial tablets [3,4,8,10,11].

In stark contrast, 3D-printed tablets produced via the Pharma Printer platform showed superior performance across all dose strengths, formulations, and sites. Gel-based CuraBlend^®^ tablets achieved mean API contents close to target (e.g., Location A 6.25 mg: 100.2%, SD 0.77%), and troche formulations, while slightly more variable, still met pharmacopeial criteria (AV < 15). Statistical analyses confirmed these observations: ANOVA showed highly significant differences (*p* < 0.001), and Games–Howell post hoc testing identified 3D-printed tablets as having consistently higher accuracy and precision compared to manually split tablets.

The accuracy and precision of 3D-printed tablets were evident in both mean API content close to target doses and low standard deviations across batches. Acceptance values were well below pharmacopeial limits in all printed groups, consistent with prior reports that 3D printing can produce highly uniform and precise dosage forms, even at low doses [22]. The reproducibility across decentralized sites further confirms that automated compounding systems can maintain quality standards independent of production location.

Beyond quality-related improvements, automated compounding offered operational advantages by enabling decentralized, on-demand production of individualized doses with digital traceability and standardized batch documentation [26,27]. Importantly, the value of the system lies not primarily in reduced preparation time, but in improved control over dosing accuracy, reproducibility, and documentation—attributes particularly relevant for precise titration in pediatric dosing and heart failure therapy, where small deviations may have significant clinical consequences.

Collectively, these findings suggest that 3D printing-based compounding offers meaningful advantages in dose standardization, traceability, and quality assurance. The technology addresses key limitations of manual dose manipulation and supports the broader shift toward individualized and precision-based dosing in hospital pharmacy practice.

Several limitations should be acknowledged. First, only one batch per condition was produced for 3D-printed tablets, and all split tablets per product were generated in a single preparation session. Consequently, the measured variability reflects within-batch (or within-session) tablet-to-tablet variation and does not capture potential batch-to-batch or session-to-session sources of variability. Treating individual tablets from a single batch as independent replicates for inferential testing introduces a risk of pseudoreplication; the statistical comparisons reported here should therefore be interpreted as supportive evidence at the tablet level rather than as definitive demonstrations of between-batch differences. Second, splitting was performed by a single technician–pharmacist pair at one site, and 3D printing was conducted at three sites but with only one batch per site; broader generalization will require multi-batch, multi-operator replication studies. Third, the study was limited to analytical quality endpoints; clinical outcomes, patient acceptability, cost-effectiveness, and workflow integration were not assessed and remain important topics for future investigation.

## 5. Conclusions

This study demonstrates that manual tablet splitting is inadequate for achieving precise and uniform dosing of metoprolol tartrate, particularly at low-dose strengths such as 6.25 mg. Despite adherence to established institutional procedures, significant variability in mass and content uniformity was observed for split tablets, with deviations that may result in clinically relevant underdosing or overdosing. These findings highlight inherent methodological limitations of tablet splitting and raise concerns regarding its suitability for reliable dose individualization in hospital practice, especially for cardiovascular medicines with narrow therapeutic windows.

In contrast, the CurifyLabs semi-solid extrusion 3D-printed tablets produced at three pharmacy locations met pharmacopeial acceptance criteria for mass variation and content uniformity in all evaluated cases. Under the conditions tested, 3D printing with the CurifyLabs Compounding System therefore produced low-dose metoprolol tartrate units with higher dose precision than manual tablet splitting. These findings are limited to the analytical comparison reported here: generalization beyond this proprietary ecosystem, and any conclusions regarding clinical outcomes, operational efficiency, or implementation feasibility, will require independent replication and prospective clinical and workflow studies that are outside the scope of the present work.

## Figures and Tables

**Table 1:** Formulation and batch summary for comparator 3D-printed conditions.

Base	Site	API (% *w*/*w*)	Target Mass (mg)	Dose (mg)	n (MV)	n (CU)
CuraBlend^®^ gel tablet	Location A	4.0	156	6.25	20	10
CuraBlend^®^ gel tablet	Location A	4.0	313	12.5	20	10
CuraBlend^®^ gel tablet	Location B	4.0	156	6.25	20	10
CuraBlend^®^ gel tablet	Location B	4.0	313	12.5	20	10
CuraBlend^®^ gel tablet	Location C	4.0	156	6.25	20	10
CuraBlend^®^ gel tablet	Location C	4.0	313	12.5	20	10
CuraBlend^®^ troche	Location A	4.0	156	6.25	20	10
CuraBlend^®^ troche	Location A	4.0	313	12.5	20	10
CuraBlend^®^ troche	Location B	4.0	156	6.25	20	10
CuraBlend^®^ troche	Location B	4.0	313	12.5	20	10
CuraBlend^®^ troche	Location C	4.0	156	6.25	20	10
CuraBlend^®^ troche	Location C	4.0	313	12.5	20	10

MV: mass variation; CU: content uniformity. All comparator batches used 4.0% *w*/*w* metoprolol tartrate. One independent batch was produced per condition (12 batches total).

**Table 2:** Content uniformity of 3D-printed metoprolol tartrate tablets (n = 10 per batch).

Base	Site	Dose	Mean (%)	SD (%)	AV
CuraBlend^®^ gel tablet	Location A	6.25 mg	100.2	0.77	2
CuraBlend^®^ gel tablet	Location A	12.5 mg	100.0	2.76	7
CuraBlend^®^ gel tablet	Location B	6.25 mg	103.4	3.26	10
CuraBlend^®^ gel tablet	Location B	12.5 mg	101.3	0.55	1
CuraBlend^®^ gel tablet	Location C	6.25 mg	98.0	0.79	2
CuraBlend^®^ gel tablet	Location C	12.5 mg	98.9	2.23	5
Troche base	Location A	6.25 mg	96.9	5.43	14
Troche base	Location A	12.5 mg	98.2	3.10	8
Troche base	Location B	6.25 mg	96.6	4.24	12
Troche base	Location B	12.5 mg	93.7	1.44	8
Troche base	Location C	6.25 mg	90.3	2.43	13
Troche base	Location C	12.5 mg	95.1	2.54	9

AV: Acceptance Value; SD: Standard Deviation. All printed batches met pharmacopeial acceptance criteria (AV < 15).

## Data Availability

The original contributions presented in this study are included in the article/Supplementary Material. Further inquiries can be directed to the corresponding authors.

## Supplementary Materials

The following supporting information can be downloaded at: https://www.mdpi.com/article/10.3390/pharmaceutics18050532/s1, Table S1a. Individual tablet mass data for split commercial metoprolol tartrate tablets (Figure 2); Table S1b. Individual content uniformity data (% label claim) for split commercial tablets (Figure 3A); Table S1c. Individual content uniformity data (% label claim) for 3D-printed CuraBlend® tablets (Figure 3B); Table S2. Stability data for 3D-printed metoprolol tartrate tablets in CuraBlend® gel tablet base; Table S3. Theoretical target mass deviation analysis for split commercial tablets; Table S4: HPLC Analytical Method Validation Summary; Table S5. In vitro dissolution profiles for 3D-printed metoprolol tartrate gel tablet formulations (Figure 3C).
