# Supplementary material for: Dose Accuracy and Content Uniformity of Low-Dose Metoprolol Tablets: 3D Printing Compared with Tablet Splitting in Hospital Pharmacy Setting
Source: Pharmaceutics. 2026 Apr 27;18(5):532. doi: 10.3390/pharmaceutics18050532 (PMC13209887; doi:10.3390/pharmaceutics18050532)
Supplement: Supplementary file 1 [file pharmaceutics-18-00532-s001.zip › pharmaceutics-4251488-supplementary.pdf]

Supplementary Material

Dose Accuracy and Content Uniformity of Low-Dose Metoprolol Tablets: 3D Printing Compared with Tablet Splitting in Hospital Pharmacy Setting

Table S1a. Individual tablet mass data for split commercial metoprolol tartrate tablets (Figure 2).

All masses in milligrams (mg). Tablets A = Mylan/Viatris; Tablets B = Rising Pharma.

Tablets A 6.25 mg (Q)	Tablets A 12.5 mg (H)	Tablets B 12.5 mg (H)	Tablets B 6.25 mg (Q)
51.3	80.3	54.7	28.3
28.1	79.5	54.2	27.2
44.0	69.8	54.9	30.6
28.2	74.8	55.6	32.9
42.8	73.1	52.2	23.7
49.8	77.2	51.4	32.9
44.2	76.2	55.9	29.6
40.3	73.2	52.9	27.8
45.1	82.5	57.2	35.8
34.6	74.6	56.3	23.4
59.5	77.6	52.7	34.6
33.6	79.6	56.0	22.5
36.2	71.4	52.9	28.8
38.0	84.3	55.4	24.7
50.8	76.2	52.2	22.2
30.5	75.4	55.7	21.9

28.0	77.4	50.8	15.9
41.2	81.6	52.9	30.2
44.4	80.8	57.2	18.9
39.5	74.3	54.3	21.2
19.3	82.8	57.5	24.9
42.7	80.0	52.6	36.0
39.4	88.6	41.4	21.8
52.0	80.3	49.5	29.8
42.1	72.9	53.0	25.7
29.8	83.2	53.2	27.7
23.5	78.3	51.5	23.7
37.0	90.0	53.1	18.5
35.3	68.5	48.8	19.7
37.5	83.2	54.5	34.0
38.0	83.5	51.9	28.3
44.3	70.7	50.6	26.3
38.6	83.6	56.3	23.3
29.9	67.8	51.3	34.9
25.9	78.4	54.6	25.1
57.9	68.0	55.0	23.9
35.0	76.3	47.0	28.3
36.9	83.7	54.2	31.1
39.7	71.1	51.8	28.5

28.7	76.1	53.2	36.0
51.3	72.9	57.8	25.2
31.9	86.1	53.3	31.8
39.5	72.2	56.1	36.7
41.6	82.8	52.7	29.9
46.4	75.3	54.1	22.5
26.2	66.0	55.9	28.9
60.3	80.3	54.4	26.3
38.7	76.1	54.3	33.9
26.9	86.2	57.0	21.6
27.7	85.9	58.3	26.9
56.1	80.6	50.0	32.5
45.0	88.5	57.7	17.9
39.0	76.3	58.1	24.4
48.7	81.4	52.8	26.6
34.9	86.8	55.5	25.5
25.3	75.4	51.3	31.1
31.4	85.8	58.2	25.3
33.1	86.6	53.1	25.8
39.7	80.0	52.5	27.1
36.8	68.1	50.9	36.6
38.0	77.7	51.0	27.5
35.9	78.7	62.2	22.8

32.8	76.6	54.8	41.7
23.6	80.4	51.6	38.8
36.8	82.3	55.7	28.7
37.4	71.3	52.8	23.6
37.6	81.3	55.7	29.7
51.8	90.0	53.3	25.6
35.1	78.5	60.6	32.1
45.3	81.4	56.7	24.8
38.8	82.2	61.0	34.1
22.8	75.0	58.4	19.8
40.4	79.8	49.9	26.1
49.2	72.2	56.4	32.1
40.5	77.4	53.4	26.6
42.6	76.7	55.1	27.9
36.0	77.2	49.5	16.2
47.8	72.9	53.7	35.3
29.3	80.5	54.1	18.5
30.8	81.7	56.8	26.3
47.1	83.6	54.8	14.9
42.9	66.8	55.9	25.0
33.1	85.3	54.2	21.7
41.5	80.0	52.5	34.1
35.1	70.6	55.5	18.5

45.4	82.1	51.8	33.7
40.3	80.1	50.0	21.4
37.4	77.6	55.8	28.0
38.1	68.5	54.8	29.7
27.7	82.0	55.5	20.7
39.5	85.3	56.2	35.0
39.4	72.1	52.8	28.6
43.8	76.2	57.4	30.2
51.0	80.0	57.0	23.3
40.5	79.5	54.4	18.5

Table S1b. Individual content uniformity data (% label claim) for split commercial tablets (Figure 3A).

#	Tablets A 6.25 mg	Tablets A 12.5 mg	Tablets B 6.25 mg	Tablets B 12.5 mg
1	64.4	68.8	113.3	90.5
2	74.2	59.0	71.4	92.6
3	63.6	67.9	93.2	87.2
4	47.4	54.9	79.4	89.6
5	40.6	64.6	69.9	89.8
6	24.9	55.8	68.7	87.3
7	68.4	62.7	49.3	77.9
8	64.3	69.7	95.3	86.4
9	85.3	57.1	59.7	86.3
10	71.6	63.0	65.1	91.0
11	67.2	68.6	109.6	92.0
12	38.3	69.3	127.6	77.4
13	68.1	70.9	98.1	99.1
14	84.1	64.9	73.3	85.1
15	70.6	60.6	94.2	86.5
16	99.2	65.9	56.5	106.3
17	67.0	65.3	79.0	86.9
18	51.5	72.4	87.7	85.4
19	67.8	70.9	83.8	78.4
20	47.7	75.2	101.2	85.2
21	105.9	56.3	82.2	83.5

22	58.8	69.3	83.5	95.9
23	61.7	64.8	89.0	97.3
24	65.9	74.2	123.1	88.3
25	88.7	72.8	92.9	91.5
26	52.3	68.2	75.3	95.8
27	38.4	75.0	142.8	87.1
28	62.9	67.7	131.7	93.5
29	60.4	69.7	96.3	87.2
30	64.7	73.7	91.5	90.5

Table S1c. Individual content uniformity data (% label claim) for 3D-printed CuraBlend® tablets (Figure 3B).

Tablets produced at three compounding sites. n = 11 units per group.

#	Methodist 6.25 mg	Methodist 12.5 mg	3551 6.25 mg	3551 12.5 mg	Saint Marys 6.25 mg	Saint Marys 12.5 mg
1	100.2	101.4	107.3	101.7	96.9	98.6
2	101.2	103.6	105.6	101.3	97.9	103.0
3	100.5	102.7	99.5	100.6	97.8	102.9
4	101.2	98.9	102.1	100.7	97.9	97.3
5	99.6	97.4	105.1	100.7	98.2	97.8
6	99.9	100.8	101.8	101.4	98.9	97.8
7	99.3	101.7	100.8	101.0	99.2	97.4
8	99.6	101.8	100.9	101.4	96.6	97.7
9	99.1	96.0	101.2	101.4	98.2	97.6
10	100.9	96.0	109.9	102.4	98.2	98.6

Table S2. Stability data for 3D-printed metoprolol tartrate tablets in CuraBlend® gel tablet base.

Long-term stability testing at 25 ± 2 °C / 60 ± 5% RH in Medi-Cup® blisters. Acceptance: assay 90–110%, pH 4.0–6.0, appearance conforms.

Formulation	Test	t = 0	1 month	3 months	6 months	9 months
0.5% w/w (+1% PS80)	Appearance	Conforms	Conforms	Conforms	—	—
0.5% w/w (+1% PS80)	Assay (%)	107.8	104.4	102.4†	—	—
0.5% w/w (+1% PS80)	pH	4.8	4.9	5.0	—	—
2% w/w (+2% PS80)	Appearance	Conforms	Conforms	Conforms	Conforms	—
2% w/w (+2% PS80)	Assay (%) (n=5)	103.5	100.5	100.7	97.2†	—
2% w/w (+2% PS80)	pH	4.9	4.9	5.1	5.1	—
3% w/w (+3% PS80)	Appearance	Conforms	Conforms	Conforms	Conforms	Conforms
3% w/w (+3% PS80)	Assay (%)	101.6	99.6	102.5	103.5	100.8
3% w/w (+3% PS80)	pH	4.6	5.0	5.0	5.1	5.1

† 0.5%: potency loss >5% by month 3. 2%: potency loss >5% by month 6.

Table S3. Theoretical target mass deviation analysis for split commercial tablets.

Deviation from theoretical half-/quarter-tablet target mass (Tablets A: 156.8 mg; Tablets B: 108.2 mg intact).

Group	n	Mean Dev. (%)	SD (%)	Outside ±10%	Mass Range (mg)
Tablets A 6.25 mg	95	−1.3	22.4	60/101 (59.4%)	19.3–60.3
Tablets A 12.5 mg	95	−0.1	7.4	17/103 (16.5%)	66.0–90.0
Tablets B 6.25 mg	95	+0.1	21.4	64/104 (61.5%)	14.9–41.7
Tablets B 12.5 mg	95	−0.1	6.2	7/98 (7.1%)	41.4–62.2

Table S4: HPLC Analytical Method Validation Summary.

Parameter	Result
Analyte	Metoprolol tartrate
Column	Waters XBridge BEH C18 (150 × 4.6 mm, 3.5 μm)
Retention Time	5.05 min
Specificity	No placebo interference; Resolution 5.81; Tailing 1.5; Plates > 111,000
System Suitability	%RSD 0.03%; Asymmetry 1.5; Plates 65,242
Linearity	r^2 = 0.9984; range 70–130 ppm
Accuracy (70%)	100.1% (RSD 0.7%)
Accuracy (100%)	99.8% (RSD 0.5%)
Accuracy (130%)	97.9% (RSD 0.1%)
Repeatability	Intra-day RSD ≤ 0.64% (3 analysts)
Intermediate Precision	Inter-day RSD ≤ 2.0%
Solution Stability	Std: +1.4%; Sample: −0.4% over 48 h
LOD	0.05 ppm (S/N 761.2)
LOQ	0.5 ppm (S/N 6135.6; RSD 1.08%)

Table S5. In vitro dissolution profiles for 3D-printed metoprolol tartrate gel tablet formulations (Figure 3C).

Dissolution in water. Drug release as % of label claim. Gel tablet formulations only; troche formulations were not subjected to dissolution testing.

Table S5a. 0.5% w/w (+1% PS80) in CuraBlend®, 400 mg (Batch B07625, n = 6).

Time (min)	% Drug Release	SD (%)
0	0.0	0.0
5	46.4	10.8
10	69.2	13.6
15	90.9	17.4
20	102.8	9.2
30	102.9	10.7
45	101.4	10.2
60	106.9	7.7

Table S5b. 2% w/w (+2% PS80) in CuraBlend®, 400 mg (Batch B07825, n = 6).

Time (min)	% Drug Release	SD (%)
0	0.0	0.0
5	36.8	7.7
10	69.9	7.5
15	77.0	3.2
20	85.5	2.8
30	102.7	8.1
45	107.1	0.5

60	108.9	0.8

Table S5c. 3% w/w (+3% PS80) in CuraBlend®, 400 mg (Batch B30623).

Time (min)	% Drug Release	SD (%)
0	0.0	0.0
5	51.5	7.3
10	85.4	9.2
15	102.5	5.0
20	105.6	1.3
30	106.3	1.9
45	105.8	0.6
60	105.7	1.2

All formulations achieved >80% drug release within 30 minutes, meeting USP specifications for immediate-release metoprolol tartrate tablets.
